# A Rare Case of Extracranial Giant Cell Arteritis in a Patient With Systemic Lupus Erythematosus

**DOI:** 10.7759/cureus.71634

**Published:** 2024-10-16

**Authors:** Oscar Vicente Vergara-Serpa, Liliana Margarita Vega Fernández, Yuleidis del Carmen Mozo Polo, Carlos Alberto Agudelo, Marta Juliana Mantilla, Sandra Pulido, Juan Camilo Santacruz

**Affiliations:** 1 Rheumatology Department, Universidad Pontificia Bolivariana, Medellín, COL; 2 Faculty of Medicine, Universidad Libre, Barranquilla, COL; 3 Faculty of Medicine, Universidad Simón Bolívar, Barranquilla, COL; 4 Rheumatology Department, Clínica las Américas Auna, Medellín, COL; 5 Rheumatology Department, Centro de Investigación en Reumatología y Especialidades Médicas (CIREEM), Bogotá, COL; 6 Rheumatology Department, Centros Médicos Colsanitas, Bogotá, COL; 7 Spondyloarthropathies Research Group, Universidad de La Sabana, Chía, COL

**Keywords:** aneurysm, giant cell arteritis, glucocorticoids, systemic lupus erythematosus, vasculitis

## Abstract

Systemic lupus erythematosus (SLE) is an autoimmune disease that affects multiple organs, and its coexistence with Giant Cell Arteritis (GCA) is extremely rare. We present, to our knowledge, the first reported case of a 56-year-old woman with SLE and extracranial GCA who presented with chest pain as the cardinal symptom. The diagnosis was subsequently confirmed by imaging studies, ruling out Takayasu arteritis and SLE-related vasculitis. She required treatment with glucocorticoids and tocilizumab, showing a satisfactory evolution. Accurate diagnosis was key to preventing serious vascular complications and achieving favorable clinical recovery.

## Introduction

Systemic lupus erythematosus (SLE) is a chronic autoimmune disease characterized by the production of autoantibodies and multisystem inflammation. It can affect multiple organs, including the skin, joints, kidneys, central nervous system, and blood vessels [1]. Studies from the last 15 years report a prevalence ranging from 9 to 241 per 100,000 person-years, and its incidence varies from 0.3 to 23.2 per 100,000 person-years, with a higher frequency in women of reproductive age [2].

The symptoms of SLE vary, with the most common being fatigue, arthritis, skin rashes, and renal involvement [3]. While vasculitis is not one of the most frequent manifestations, it can occur and predominantly affect small-caliber vessels (SCV), contributing to the clinical heterogeneity of the disease [4]. Although large-vessel vasculitis (LVV) is rare in SLE, it has been reported in some cases, generally associated with more severe disease and a poor prognosis, including serious complications such as infarctions, aneurysms, or thrombosis [5].

On the other hand, giant cell arteritis (GCA) is a granulomatous vasculitis that primarily affects large- and medium-sized arteries, especially the branches of the aorta [6]. The classic symptoms include headache, jaw claudication, scalp tenderness, and, in some cases, blurred vision or sudden vision loss due to retinal ischemia [7]. GCA is more common in individuals over the age of 50, with an incidence ranging from 7 to 29 cases per 100,000 person-years, and it is more prevalent in populations of European descent. It predominantly affects women, with an average onset age of 70 years [8].

The coexistence of SLE and GCA in a single patient is extremely rare, presenting diagnostic and therapeutic challenges due to the overlapping clinical manifestations and the different therapeutic approaches required for these two diseases. Here, we present the case of a patient with a prior diagnosis of SLE who developed a condition consistent with GCA, manifested by chest pain and findings of large-vessel vasculitis, including aneurysmal dilation of the ascending aorta and axillary artery stenosis.

## Case presentation

A 56-year-old female teacher, residing in Medellín, Colombia, with a diagnosis of SLE since 1985, which initially presented with thrombocytopenia, Anti-DNA 1:160, ANA (AC-4) 1:160, hypocomplementemia, sensory polyneuropathy, and transverse myelitis, leaving her with sequelae of neurogenic bladder and chronic neuropathic pain in her left lower limb. She was under outpatient treatment with prednisolone 5 mg/day, hydroxychloroquine 200 mg/day, gabapentin 600 mg every 8 hours, and duloxetine 60 mg/day, the latter two for chronic pain management, and tolterodine 4 mg/day. She also had a family history of a sister with SLE.

On December 26, 2023, she presented with a five-day history of sudden, constant chest pain of atypical characteristics. The pain did not radiate, lacked pleuritic features, and was associated with palpitations, sweating, and fatigue. Additionally, she reported neck pain localized to the right anterior lymph node chain. She denied dyspnea, arthritis, myopathy, cough, fever, mucocutaneous symptoms, gastrointestinal, genitourinary, neurological symptoms, and/or Raynaud's phenomenon.

The patient was hospitalized for further evaluation. During her stay in the emergency department, her heart rate was recorded at 88 bpm, blood pressure in the right upper limb was 160/95 mmHg, and in the left upper limb, it was 100/95 mmHg. Her respiratory rate was 18 bpm, her body temperature was 36.2°C, and her body mass index was 26.8 kg/m². Physical examination revealed hypoesthesia in the right lower limb, with no signs of focal neurological deficits. No pleuropericardial rubs, carotid bruits, or aortic murmurs were detected, and pulses were not reduced. It was decided to investigate for possible abnormalities suggesting the involvement of cardiopulmonary structures and/or the aorta and its branches (Table 1).

**Table 1:** Admission cardiac and pulmonary imaging studies

Test	Result
Chest X-ray (12/26/2023)	Findings: - Normal cardiac silhouette - Elongated aorta - No pathological mediastinal widening - Normal vascular pedicle - Normal pulmonary circulation - Patent tracheobronchial tree - No acute parenchymal processes - Fine reticular opacities (incipient interstitial involvement) - Costophrenic and cardiophrenic angles clear - Degenerative changes in the thoracic spine. Impression: Negative for acute pleuropulmonary process.
Electrocardiogram (12/26/2023)	Findings: - Sinus rhythm - Heart rate: 88 bpm - No AV block - No bundle branch block - No supraventricular extrasystoles - No signs of pulmonary hypertension.
Echocardiogram (12/28/2023)	Findings: - Normal left ventricle size, normal mobility, concentric remodeling of wall thickness - Ejection fraction: 65% - MAPSE: 13mm - Right ventricular TAPSE: 18mm (normal contractility) - Normal atria - Mitral valve: Calcified nodule on anterior leaflet - Aortic valve: Mild sclerosis, very mild insufficiency - Pulmonary valve: Normal - Tricuspid valve: Mild insufficiency - Thoracic aorta: Normal diameters, normal wall appearance - Estimated pulmonary artery systolic pressure (PASP): 26mmHg - Tricuspid regurgitation velocity: 229 cm/s

The laboratory tests revealed an increase in acute phase reactants (APR), elevated aminotransferases, anemia, and thrombocytosis. Troponin was negative (Table 2). 

**Table 2:** Admission laboratory tests ESR: Erythrocyte sedimentation rate; CRP: C-reactive protein; MCV: Mean corpuscular volume; MCH: Mean corpuscular hemoglobin; AST: Aspartate aminotransferase; ALT: Alanine aminotransferase; Anti-DNA: Anti-double stranded DNA antibodies; ENAs: Extractable nuclear antigens; C3-C4: Complement; VDRL: Venereal disease research laboratory test (non-treponemal manual test); HIV: Human immunodeficiency virus 1 and 2 (ELISA TEST)

Test	Result	Reference Values
ESR	97 mm/h	(< 20 mm/h)
CRP	15.7 mg/dL	(< 5 mg/dL)
Ferritin	972 ng/mL	(12 - 150 ng/mL)
Hemoglobin	11 g/L	(12 - 15 g/L)
Hematocrit	33%	(35 - 42%)
MCV	87 fL	(80-100 fL)
MCH	30 pg	(27 - 33 pg per cell)
Platelets	618,000/mm³	(150,000 - 450,000/mm³)
Troponin	Negative	N/A
Urinalysis	pH 8, Specific gravity 1010, Proteins 0 mg/dL, Normal sediment	N/A
AST	70 U/L	(0 - 40 U/L)
ALT	121 U/L	(0 - 41 U/L)
Anti-DNA	Negative	N/A
ENAs	Negative	N/A
C3	128 mg/dL	(88 - 201 mg/dL)
C4	16 mg/dL	(15 - 45 mg/dL)
VDRL	Non-reactive	N/A
HIV	Negative	N/A
Hepatotropic Viruses	Negative	N/A

**Figure 1:**
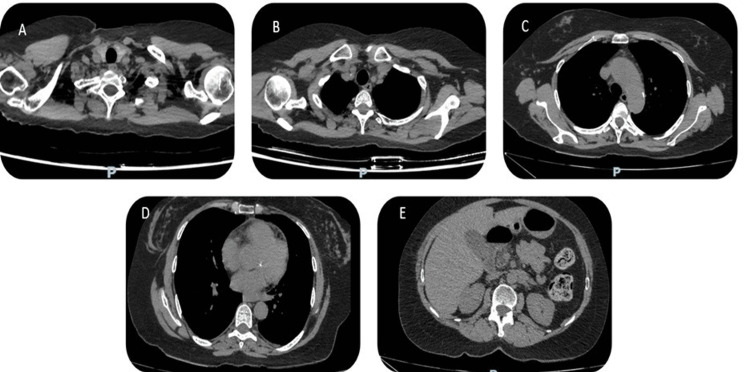
Chest CT scan Image A (upper section): visualization of the right subclavian artery showing signs of stenosis in its distal portion, just at the exit of the quadrilateral space. Image B (middle section): shows aneurysmal dilation of the ascending aorta with a diameter of 44 mm. The aortic walls exhibit regular circumferential thickening greater than 3 mm, indicative of aortitis. Image C (lower section): Thickening and enhancement of the descending aortic wall, suggesting large-vessel vasculitis, compatible with giant cell arteritis. Image D (thoracic view): Evidence of thoracic aorta dilation with involvement of the aortic arch. No signs of thrombosis. Image E (Abdominal section): Evaluation of the abdominal system, with no evidence of aneurysmal dilation or significant stenosis in the main abdominal vessels.

**Figure 2:**
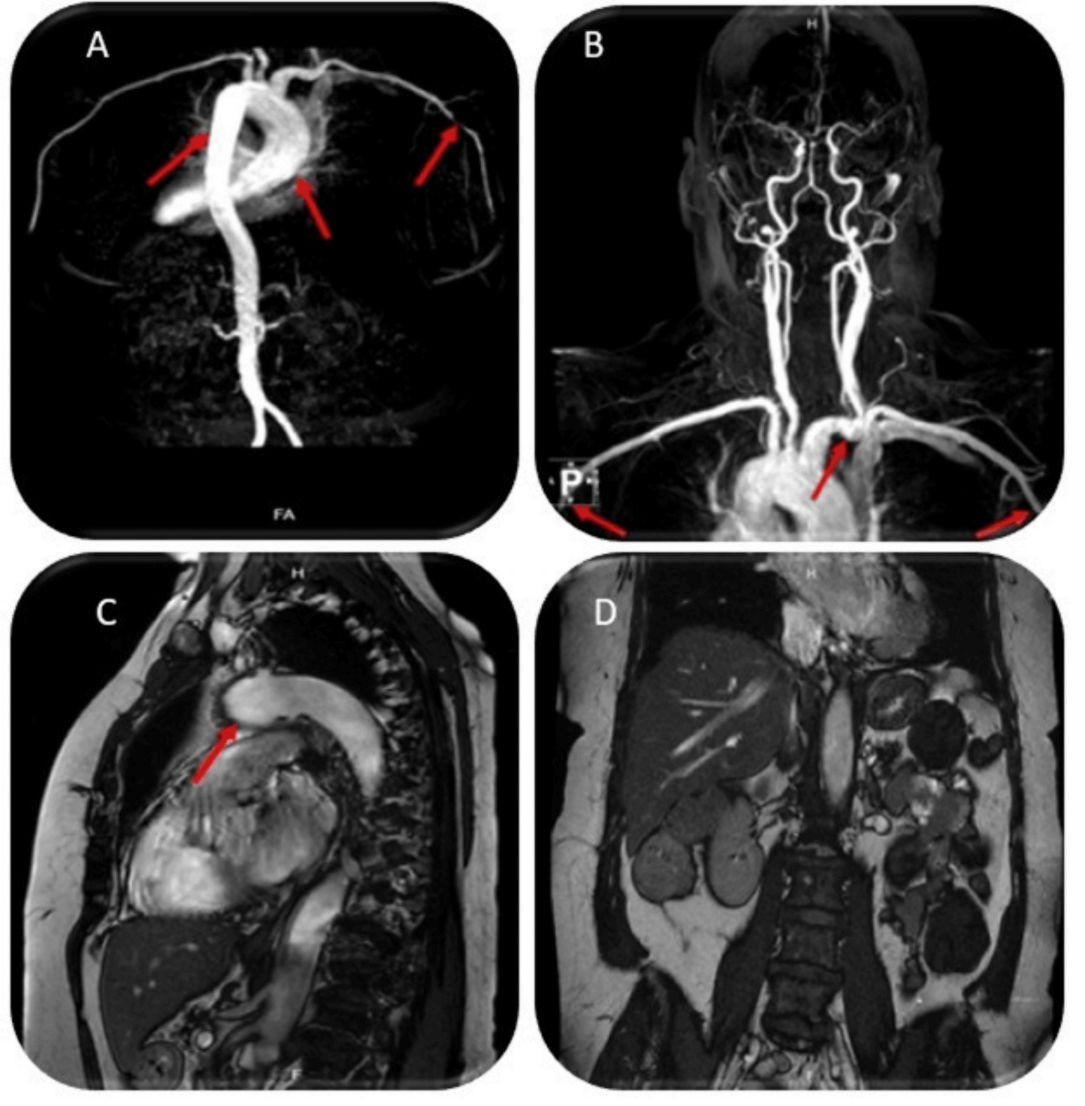
MR angiography of the neck, chest, abdomen, and pelvis Image A: Significant dilation of the ascending aorta with thickening of the aortic wall (red arrow). Image B: The carotid and vertebral arteries show no evidence of stenosis, obstruction, or flow alterations. Stenosis of the distal right axillary artery at the level of the quadrilateral space, aneurysmal dilation of the ascending aorta, and enhancement of the descending aortic wall are represented in fluid-sensitive sequences (red arrow). Image C (sagittal view): Aneurysmal dilation of the ascending aorta along with mild pericardial thickening (red arrow). Image D: Normal evaluation of the abdominal system.

LVV was suspected based on the findings. A Doppler ultrasound of the temporal and axillary arteries was performed, and abnormalities in the flow of the right axillary artery (RAA) were reported. An MR angiography was conducted to better characterize the arteries, revealing stenosis of the distal right axillary artery, aneurysmal dilation of the ascending aorta, and enhancement of the descending aortic wall, suggestive of LVV (Figures 1, 2). 

Given the findings, a differential diagnosis was proposed between GCA, late-onset Takayasu Arteritis (TAK), or SLE-associated vasculitis. However, the patient's age (between 50 and 60 years) posed a diagnostic challenge. Using the 2022 EULAR criteria [9,10], she did not meet the classification criteria for either GCA or TAK. The clusters proposed by Gribbons et al. [11] were applied, placing the patient in "Cluster 6," with an 85.5% probability of GCA and a 14.5% probability of TAK. SLE-related vasculitis was ruled out due to the absence of clinical and laboratory activity (SLEDAI 2K=0).

Finally, extracranial GCA was diagnosed. The patient started treatment with glucocorticoids (methylprednisolone 500 mg/day for three days), followed by prednisone 50 mg/day, with a reduction of 10 mg every two weeks until reaching a minimum dose of 10 mg. On the third day, 162 mg of subcutaneous tocilizumab was administered, and the same dose was prescribed at hospital discharge.

The patient was discharged after five days with significant improvement in acute phase reactants, aminotransferases, anemia, and thrombocytosis. At six months follow-up, she was asymptomatic.

## Discussion

The coexistence of SLE and GCA is extremely rare and presents a diagnostic challenge due to overlapping symptoms and the differences in the pathophysiology of both diseases. SLE is a multisystem autoimmune disease characterized by the production of autoantibodies and predominantly affects small-caliber vessels [4], whereas GCA is a granulomatous vasculitis primarily affecting large-caliber arteries [6]. In this case, the patient presented with manifestations consistent with both conditions, complicating the initial diagnosis.

In patients with SLE, chest pain is a relatively common symptom and can have multiple causes. The most common etiologies include pericarditis, myocarditis, and coronary artery disease, all related to inflammation of cardiac tissues and vascular complications of SLE [12]. Other causes include pulmonary embolism, lupus pneumonitis, and pleural involvement [13]. However, in this case, the absence of pleuropericardial involvement and atypical vascular findings (aneurysmal dilation of the aorta and vascular wall thickening) pointed toward a different cause of chest pain. In the study by Masoom Modi et al., where the prevalence and causes of chest pain in SLE patients were determined, only 7.2% of cases were attributed to cardiovascular disease (infarction, angina, or microvascular disease), and 3.1% to pericarditis, highlighting the prevalence of non-cardiac causes [14].

The diagnosis of GCA was supported by imaging studies. MR angiography showed aneurysmal dilation of the ascending aorta and stenosis of the distal axillary artery, indicating significant involvement of large vessels. Additionally, Doppler ultrasound of the RAA revealed abnormalities in distal flow, further supporting the diagnosis. These findings allowed the avoidance of a temporal artery biopsy, as imaging criteria and high clinical suspicion are sufficient to confirm the diagnosis [15]. Furthermore, according to the European League Against Rheumatism (EULAR) recommendations, in patients with high clinical suspicion and positive imaging studies, a biopsy is not necessary to confirm the diagnosis [16].

TAK was ruled out for several key reasons. First, the patient’s age (57 years), as it mainly affects women under 40. Moreover, although TAK can involve large arteries such as the aorta and its branches, the pattern of involvement in this patient, which included aneurysmal dilation of the ascending aorta and axillary artery involvement, was more consistent with GCA than TAK [10]. Cluster analysis also supported this conclusion, indicating a significantly higher probability of GCA (85.5% vs. 14.5%) [11]. Finally, the typical clinical criteria for TAK, such as limb claudication, pulse discrepancies between limbs, and involvement of the abdominal aorta with renal and/or mesenteric arteries, were not found in this patient [10].

SLE-related vasculitis was ruled out due to the absence of clinical and laboratory activity. Although SLE can cause vasculitis, it usually affects small and medium-sized vessels, and large vessel involvement, such as the aorta, is extremely unusual [4]. In the systematic review by Akiyama M et al., aortitis in SLE primarily affected the ascending aorta (60% with thickening, 20% with aneurysm). Symptoms are nonspecific (fever, dyspnea, chest pain) with elevated CRP and ESR levels. Additionally, all patients showed elevated ANA, anti-dsDNA, and/or anti-Smith antibody titers [5]. The patient was in prolonged remission of her disease, making an SLE flare less likely as the cause of the current presentation.

The management of GCA in this patient included the use of high-dose glucocorticoids and the introduction of tocilizumab, an interleukin-6 (IL-6) inhibitor that has proven effective in treating refractory GCA or GCA with a high risk of complications [17]. Additionally, tocilizumab has shown promise as a therapeutic option in the treatment of SLE, according to recent evidence in various clinical scenarios [18-20].

The combination of these treatments led to rapid clinical improvement, with normalization of APR and stabilization of vascular findings. At six month follow-up, the patient was asymptomatic, reinforcing the effectiveness of the implemented management.

During the systematic literature search, it was identified that this is the first documented report worldwide of the coexistence of SLE and extracranial GCA. The only previous report was published in 1988 by Bunker CB et al., describing a case of SLE coexisting with GCA but with cranial involvement [21].

This case highlights the importance of a multidisciplinary approach and the use of advanced diagnostic tools to identify the underlying pathology in patients with complex autoimmune diseases. The accurate identification of LVV, along with early and adequate treatment, is crucial to prevent long-term complications, such as aneurysm progression or ischemic events.

## Conclusions

In conclusion, the case illustrates a rare association of GCA in a patient with SLE, highlighting the need to consider broad differential diagnoses. The current clinical presentation of this case report classifies it as a GCA with large vessel involvement superimposed on a cranial GCA. Chest pain could be an important symptom to establish large vessel involvement. Our case is consistent with what has been described in the literature since GCA with large vessel involvement tends to occur in younger patients with a lower probability of reporting headache or claudication in the upper extremities. Early treatment with tocilizumab may prevent serious vascular complications such as aneurysms or aortic dissection. This treatment would greatly help rapidly reduce the glucocorticoid dose, which is already high in patients with SLE, thus reducing the cumulative damage induced by this medication.
